# Characterizing Long Interval Cortical Inhibition over the Time-Frequency Domain

**DOI:** 10.1371/journal.pone.0092354

**Published:** 2014-03-18

**Authors:** Luis Garcia Dominguez, Natasha Radhu, Faranak Farzan, Zafiris J. Daskalakis

**Affiliations:** Temerty Centre for Therapeutic Brain Intervention, Centre for Addiction and Mental Health, University of Toronto, Toronto, Canada; University Medical Center Goettingen, Germany

## Abstract

**Objective:**

Long-interval cortical inhibition (LICI) can be recorded from motor and non-motor regions of the cortex through combined transcranial magnetic stimulation (TMS) with electroencephalography (EEG). This study aimed to evaluate additional dimensions of LICI characteristics over an extended time-frequency and spatial domain. This was done by introducing two alternative measures of LICI signal amplitude: the Discrete Fourier Transform (DFT) and the Hilbert transform (HT). Both approaches estimate signal amplitude not taking into account the phase. In both cases LICI was measured as the difference between the unconditioned and conditioned activity evoked by the test pulse. Finally, we evaluated whether the topographical patterns of single and paired responses differed beyond the expected variations in amplitude.

**Materials and Methods:**

LICI was delivered as single and paired pulses to the motor cortex (MC) and dorsolateral prefrontal cortex (DLPFC) in 33 healthy subjects with TMS-EEG.

**Results:**

Significant differences (p<0.0001) between the unconditioned and conditioned evoked activity were found for both the DLPFC and MC using both methods (i.e., DFT and HT) after correcting for multiple comparisons in the time-frequency domain. The influence of inhibition was found to be significantly larger in space and time than previously considered. Single and paired conditions differ in intensity, but also in their topographic pattern (i.e., the specific spatiotemporal configuration of active sources).

**Conclusion:**

Similar results were found by both DFT and HT. The effect of inhibition across the cortex was also found to be complex and extended. In particular, it was found that LICI may be measured with high sensitivity in areas that were relatively distant from the stimulation site, which may have important practical applications. The analysis presented in this study overcomes some limitations of previous studies and could serve as a key reference for future studies examining TMS-indices of inhibition/excitation in healthy and diseased states.

## Introduction

Long-interval cortical inhibition (LICI) refers to the transient suppression of neuronal activity when a suprathreshold transcranial magnetic stimulation (TMS) test pulse is preceded by a second suprathreshold TMS conditioning stimulus using an interstimulus interval between 50 to 200 ms. This paradigm results in the inhibition of the motor evoked potential (MEP) produced by the test stimulus [1], [2]. This phenomenon is closely associated with GABA_B_ receptor-mediated inhibitory neurotransmission. This is because the duration of LICI is comparable to the slow inhibitory postsynaptic potentials (IPSPs) [3]–[5]. Additionally, LICI is potentiated by GABA_B_ receptor agonists (e.g., baclofen) as documented by numerous lines of evidence from pharmacological studies [6], [7]. Finally, LICI is activated by high intensity suprathreshhold paired-pulse stimuli that is similar to the high activation thresholds needed to activate GABA_B_ inhibitory IPSPs [8].

More recently, through the combined use of interleaved TMS with EEG, LICI can be measured from non-motor regions of the cortex (e.g., dorsolateral prefrontal cortex (DLPFC)) [9]–[12] in which there is a suppression of the TMS-evoked potential (TEP) over the site of the stimulation when a conditioned TMS pulse precedes a test TMS pulse by 100 msec. While the effect of inhibition is particularly strong and easy to document from MEP recordings using simple measures, the TEP is more challenging due to its lower signal to noise ratio.

Previous studies from our lab have restricted the assessment of LICI to the electrode closest to the stimulation site and to the first 100–150 ms. This approach has been successful at documenting LICI in the normal brain as well as LICI differences in clinical populations [13], [14]. It is also consistent with a model of LICI that assumes that the conditioned response is a fraction of the unconditioned one, and that, this fraction (expressed as percent of inhibition), tends towards one (0% inhibition) as time and space unfolds (the characteristic profile in frequency however, has been shown to be variable across different studies, and it is generally not assumed to be homogeneous). While both, the local action of the TMS pulse over the high-threshold inhibitory interneurons, and the characteristic temporal course of the IPSP induced by GABA_B_ offers good support to this model, the nonlinear nature of the brain and the heterogeneity in connectivity pathways across the cortex can potentially reveal additional features of LICI that have not been explored. In the context of complex non-linear systems, of which the brain is a characteristic element, it is often emphasized that some properties of the whole cannot be reduced to the sum of their components, the so called “emergent” properties. In particular, it is well known that the response of a non-linear dynamical system to a small perturbation can lead to a large scale unpredictable pattern. That inhibition fades away in time and space is a linear extrapolation resulting from a local analysis that may or may not hold over the entire spatial and temporal-frequency domain. Therefore, the study of the characteristics of LICI over an extended parameter domain is the subject of our analysis.

Past results on LICI analyses revealed evidence for inhibition over a restricted domain, by sacrificing either, the temporal, the spatial or the frequency component. Examples are: the fixed window analysis [9], [11], the measure of peak amplitude [12] and the analysis over a 25 ms sliding window [15] at a single 1–40 Hz bandpass. There is no comprehensive study that examines the entire time-frequency and spatial domain including baseline EEG activity. One of the reasons for this is that by presenting a multidimensional analysis there is an increased likelihood of committing Type I error due to the problem of multiple comparisons [16], [17].

To explore LICI's spatiotemporal dynamics new tools have to be applied, specifically a time-frequency analysis of some measure of signal amplitude, since LICI has to be defined as a decrease of the “activity” (in some of its flavours: amplitude, power, spectral density, etc.) evoked by the conditioned test stimulation in comparison to the normal evoked unconditioned activity. A natural choice to this purpose is the Discrete Fourier Transform (DFT) analysis over a sliding time window. Along with this measure we would also introduce a complementary analysis that can potentially offer more flexibility and better temporal resolution. This alternative analysis is the amplitude of the discrete-time analytic signal computed via the Hilbert transform (HT).

Two different areas of stimulation are explored in this study (i.e., the left dorsolateral prefrontal cortex (DLPFC) and the left motor cortex (MC)). These are two previously characterized areas in relation to LICI. The DFT would be calculated using a custom tool included in the EEGLab package [18] called “Event Related Spectral Perturbation” (ERSP). This is a standard measure of evoked activity in the time-frequency domain which has been initially motivated and developed in relation to cognitive paradigms. In the context of TMS, ERSP was also used in [19], to discriminate groups based on the response to single-pulse TMS. The analytic signal approach, which is a less common tool in the field of ERP/TEP analysis, would also offer a similar characterization of signal power involving fewer parameters, since there is no need to specify a temporal window. Their combination would present a more general view of the event-related changes that are caused by the inhibitory mechanisms probed by the classical TMS paired-pulse protocols.

The time–frequency analyses that are introduced here overcome some limitations of previous methodologies in the assessment of LICI. In particular, we can estimate two key features, the period influenced by inhibition and the strength and location at the peak of the inhibition. The period or duration can be simply documented by inspecting at what time point the value of amplitude differences ceases to be statistically significant for each particular frequency. This is possible due to the fact that a pre-determined fixed temporal window does not limit the analysis. The location and strength can also be estimated because we have chosen an analysis that can be evaluated at any time point, since it is not dependent on particular temporal features such as peaks and it is not affected by the phase of the oscillation, which is a problem with any description of the TEP in the time domain. For example, a phase shift between two oscillations (two scalar time series) can affect their comparisons in amplitude, principally when the period of the oscillation is large in relation to the window of analysis. However, since both the DFT and HT produce a complex set of coefficients, phase can be effectively removed leaving the amplitude (or power) as the only sufficient descriptor.

In this study we aimed to present a spatial, time-frequency characterization of LICI that is intended to be as free from domain constraints as possible. This approach offers a comprehensive multidimensional picture of LICI and its consequences over the whole brain cortical network. In addition to this, a novel approach is presented to disentangle magnitude and pattern from the response to the test TMS pulse. The reason for this is that indexing LICI by amplitude differences alone is a simplification that can hide interesting spatial details about the differences in specific configuration of source activation within the brain in the single and paired conditions. Considering the brain as a complex non-linear system, inhibition should not only affect the magnitude of the response but also the circuitry involved in the response. In other words, the conditioned response should not only be quantitatively, but also qualitatively different to the unconditioned one.

## Materials and Methods

Thirty-three volunteers participated in the study. Participants were free of any neurological and psychiatric disorders as confirmed by history and the Structured Clinical Interview for the Diagnostic and Statistical Manual for Mental Disorders –IV. All experimental procedures were approved by the Centre for Addiction and Mental Health in Toronto, Canada, in accordance with the Declaration of Helsinki. All subjects gave their written informed consent.

Monophasic TMS pulses were administered using a 7 cm figure-of-eight coil, and two Magstim 200 stimulators (Magstim Company Ltd, UK) connected via a Bistim module. TMS was administered over the left motor cortex and DLPFC. We evaluated LICI at the optimal 100 ms interstimulus interval [8]. One hundred TMS stimuli were delivered per-condition (paired and single-pulse) every 5 seconds. The intensity of TMS pulses was determined at the beginning of each experiment and it was set such that it elicited an average motor evoked potential of 1 mV peak-to-peak upon delivery of 20 pulses over the motor cortex. Both the conditioning stimulus and test stimulus were delivered at the same suprathreshold intensity.

### Localization of the Motor Cortex

The TMS coil was placed at the optimal position for eliciting motor evoked potentials from the right abductor pollicis brevis muscle, which corresponded to a region between the electrodes: FC3 and C3.

### Localization of DLPFC

Localization of DLPFC was achieved through neuronavigation techniques using the MINIBIRD system (Ascension Technologies) and MRIcro/registration software using a T1-weighted MRI scan obtained for each subject with seven fiducial markers in place [9], [13].

### EEG recording and pre-processing

EEG was acquired through a 64-channel Synamps 2 EEG system. A 64 channel EEG cap was used to record the cortical signals, and four electrodes were placed on the outer side of each eye, and above and below the left eye to closely monitor eye movement artifacts. All electrodes were referenced to an electrode positioned posterior to the Cz electrode. This location is in the area least contaminated by muscle activity [20] and is one of the most used reference montages in the literature. EEG signals were recorded DC and with a low pass filter of 100 Hz at a 20 kHz sampling rate, shown to avoid saturation of the amplifiers and minimize the TMS related artifact [9], [10].

The TMS-EEG signals, MC and DLPFC, were processed offline using MATLAB (The MathWorks Inc. Natick, MA, USA). All signals were down sampled from 20 kHz to 1 kHz and segmented with respect to the test stimulus such that each epoch included 1000 ms pre-stimulus baseline and 1000 ms post-stimulus activity. Epochs were baseline corrected with respect to the TMS-free pre-stimulus interval.

A semi-automatic multistep protocol for removal of numerous artifact of independent nature was implemented. Every step in which visual inspection has to take place in order to judge channels or components to remove, was optimized so that the whole dataset could be inspected over a few hours, with minimal subjective intervention. All trials were visually inspected and then sorted according with different criteria (features), kurtosis, standard deviation and low and high-frequency power across all its channels. The sorted trials across all subjects were marked for rejection by visual inspection. To ensure consistent criteria of trial rejection the actual rejection only took place by using linear discriminant analysis over the feature space. The discriminant function was fitted over the set of trials marked and unmarked for rejection and the rejection was only effected over the new labeling imposed by the discriminant function. Within the remaining trials bad channels were also removed based on a similar procedure, as well as visual inspection for abnormal activity. Time windows containing TMS pulses were removed, specifically the window that extend from the first TMS to 10 ms after the second TMS in the paired condition, as well as the same time window in the single condition although this one did not contain the conditioning TMS. That is, if trials are aligned with 0 at the start of the test-TMS, the actual time range removed in all trials was [−100 ms 10 ms]. Then Independent Component Analysis (ICA) was applied in order to remove a specific component that is TMS-related, a slow exponential decay than can last a few milliseconds to more than 100 ms in some subjects. To this end Fastica [21] was run over the dataset and the result for each participant was subject to visual inspection after ordering first the components that were most likely to capture the decay. The identified components were removed and the data was digitally filtered by using a zero-phase shift 1–100 Hz band pass filter. The 60 Hz powerline artifact was removed from each trial across all channels by using the Thomson *F*-test based on multitaper spectral estimate techniques [22]. A second run of ICA was applied in order to eliminate eye blinks, eye movement, as well as components with unusually elevated high frequency spectra. For each specific artifact, components across all files were ordered according to specific features that capture their likelihood to be the specific artifact. Different artifacts where captured by different features, for example, a correlation to a topological template, or a statistic computed from the columns of the mixing matrix. In this ordered dataset visual inspection determined whether the component has to be removed. After these steps, removed channels were interpolated and the data was transformed to current source density to improve spatial resolution.

### Discrete Fourier Transform

The DFT was calculated via “Event related spectral perturbation” (ERSP) using the newtimef() function in the open source toolbox EEGLAB version 11.044 [18]. The function was applied to the average trial. A period from 1 sec before the test TMS to 1 sec after was analyzed using two-hundred equally spaced sliding widows of size 256 ms and twenty-two frequencies equally spaced in the range 4 to 50 Hz. The baseline activity was subtracted and the ERSP was expressed in dB as 10*log10(R) where R is the ratio of power between the signal and its baseline period. ERSP was computed for each EEG sensor (60) within each condition (4) (conditioned and unconditioned DLPFC (cDLPFC & uDLPFC), and conditioned and unconditioned MC (cMC & uMC) on each participant (33). The analysis yielded an ERSP matrix whose dimension is [200×22×60×4×33] (i.e. times, frequencies, channels, conditions, participants), 34,848,000 values in total.

### The Hilbert Transform

The analytic signal (*S*) can be understood as the complex representation of any real value time series (*s*). It is expressed as 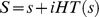 where 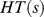 is the Hilbert transform of s. The Hilbert transform is the original real sequence *s* after a 90° phase shift at each frequency. The obtained analytical series has the same amplitude and frequency content as the original real data. The motivation for computing the HT in this study is that the analytical signal contains a measure of the instantaneous amplitude of the original series that is independent of the phase of the oscillation. Intuitively the HT amplitude corresponds to the envelope amplitude of any narrow-band signal.

For each subject, channel, and condition the average signal across trials was band-filtered at the same central frequencies (f*_i_*) as in the ERSP analysis. The filter consisted in a Finite Impulse Response (Matlab fir1 function) with cutoff frequencies f*_i_* ± 2 Hz. From these filtered signals the average across trials was computed followed by the Hilbert transform (HT). From the complex signal obtained the phase was removed and the power was calculated, in analogy with the ERSP power, as the 10*log10(R) where R is the ratio of the Hilbert amplitude between the signal and its baseline period. As we already stated, this quantity, unlike the case of the ERSP, is instantaneous. After performing the transformation the period between −700 to 800 ms was retained and the rest discarded, this period correspond to 1500 time points. The analysis yielded a matrix of amplitudes whose dimension is [1500×22×60×4×33], 261,360,000 values in total.

### Statistical Analysis

#### Strength and size of inhibition

LICI is estimated as the power spectral density (DFT) or analytical signal amplitude (HT) (both scaled to dB from baseline) evoked by the unconditioned stimulation minus the same parameter for the conditional stimulation. We will denote this parameter as LICI^DFT^ or LICI^HT^, depending on the method used in its calculation. This definition is closely related with the previous assessments of LICI in which this quantity is estimated as a percent from a ratio between the conditioned and conditioned evoked activity. In fact, by evaluating the difference between two logarithmic quantities we are evaluating the logarithm of a ratio (i.e. log(a) − log(b)  =  log(a/b)). Although the logarithmic transformation was not used in the original studies it is introduced here to enforce a normal distribution of values.

After averaging across channels, time-frequency maps (TF-map) of DFT and HT were also obtained for each participant for each of the four conditions cDLPFC, uDLPFC, cMC and uMC. These TF-maps were then subject to statistical hypothesis testing.

#### Correcting for multiple comparisons

In order to control for multiple comparison (in the time-frequency space) we used the cluster mass test for within subject conditions, a nonparametric statistical test described in [17] that was adapted to our specific circumstances. The method relies on using relevant clustering of adjacent time-frequency samples to reduce the number of comparisons to one. In order to explain the method, assume we take only data from the motor paradigm and would like to compare the (U (unconditioned) paradigm to the C (conditioned) paradigm. This involves the following steps:

1. The baseline of every TF-map is removed. Leaving two 3D matrices (U and C) of post stimulus time, with the 22 frequencies and 33 subjects.
2. For each sample in the time-frequency domain calculate a one-tailed (U>C) paired t-test across the 33 subjects.
3. Select all samples whose t-scores are larger than those at alpha  =  0.05.
4. Cluster the selected samples in connected sets on the basis of time-frequency adjacency and sum all t-scores in the cluster. If there is more than one cluster select the one with the greatest sum. This sum would be designed as S^0^.
5. Repeat steps 2-4 after randomly permuting conditions within each subject 10,000 times. That is, each time, in some randomly taken set of subjects the U condition would be in its C condition and vice versa. The new sums would be denoted as S^k^ where k = 1…10,000. There is a 50% chance of finding permuted conditions at each iteration, in each subject.
6. Calculate the proportion of random permutations that resulted in a larger test statistic than the observed one. This proportion is the p-value.

According to this protocol we are specifically testing whether the U-condition is significantly bigger than the C-condition globally across the entire TF domain.

Point 4 in the above description uses t-scores as an index of the strength of inhibition, since the magnitude of t-score is a measure of how different the responses are to both single and paired stimulations after correcting for the dispersion of the data. However, strength can be evaluated using a different statistic, for example, raw differences or the total area of the cluster. This total area would also be assessed in this paper to show that inhibition is also large in its extension over the time-frequency domain.

#### Pattern differences

**Figure 1:**
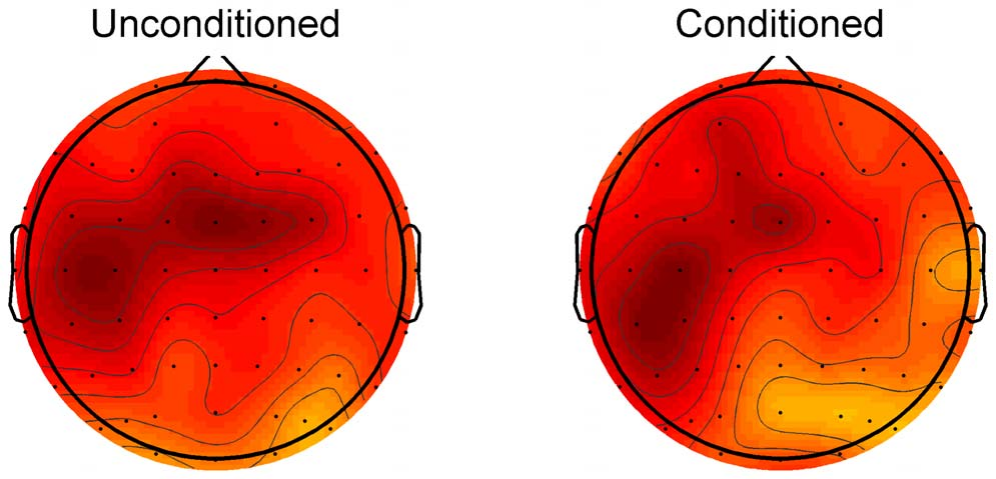
Topographic maps of Hilbert amplitudes in dB from stimulation to the motor cortex. The maps are obtained at 100±2 Hz band. Values are averages across participants.

A test on dissimilarity between spatial patterns (i.e. topographic maps) would be introduced. Consider figure 1, the topographic map on the left is the unconditioned response at 10.5 Hz and time 100 ms, the one on the right is the conditioned response at the same time and frequency. The question is, are these patterns (statistically) the same? These patterns are a direct consequence of the specific configuration of source activation within the brain, and we would like to compare them with statistical confidence. We proceed this time by introducing some variations on the cluster mass test explained above. First, consider that a pattern is fully defined by the collection of all topographic values, 60 in total, corresponding to the same amount of electrodes. That is, a pattern is a 60-dimensional vector. Comparing two patterns (U and C-topographic plots) is just applying a measure of distance between two of these vectors. However not every measure of distance is appropriate to compare patterns. For example the common Euclidean distance can involve both, a difference in direction as well as a difference in the magnitude of the vector (the pattern intensity). To disentangle both contributions we can use the included angle between the vectors (Ω), which is not affected by magnitude variations, only by movements of the relative rotation between the vectors. Thus, we identify a pattern by the direction and not the amplitude of the 60-dimensional vector. Correspondingly, by using Ω or the more computationally efficient 1-cos(Ω) we are effectively estimating pattern differences. This parameter (1-cos(Ω)) grows monotonically when one electrode is rotated away from the other in any direction, but is not affected by changes in vector's amplitude. The parameter is 0 when both vectors point in the same direction, 1 when they are perpendicular, and 2 when they point in opposite directions. With this measure of pattern difference we can simply find the proportion of random partitions that resulted in a larger value of the parameter (p-value). This time we proceed by introducing a modification to the algorithm detailed above. In point 3) instead of thresholding the data by a paired t-test we do it non-parametrically by using the same procedure of random permutations. That is, a time-frequency pair is considered significant if it is bigger than 99% of the same sample in the population of random permutations. The reason for this modification is that the values of (1-cos(Ω)) are restricted to the interval [0 2] and may deviate substantially from a normal distribution. The rest of the test proceeds the same way as described above. In short, a randomization of conditions is used first to calculate the clusters of significant values of the original population followed by a similar procedure but, this time, to calculate the likelihood of the sum within the biggest cluster.

## Results

**Figure 2:**
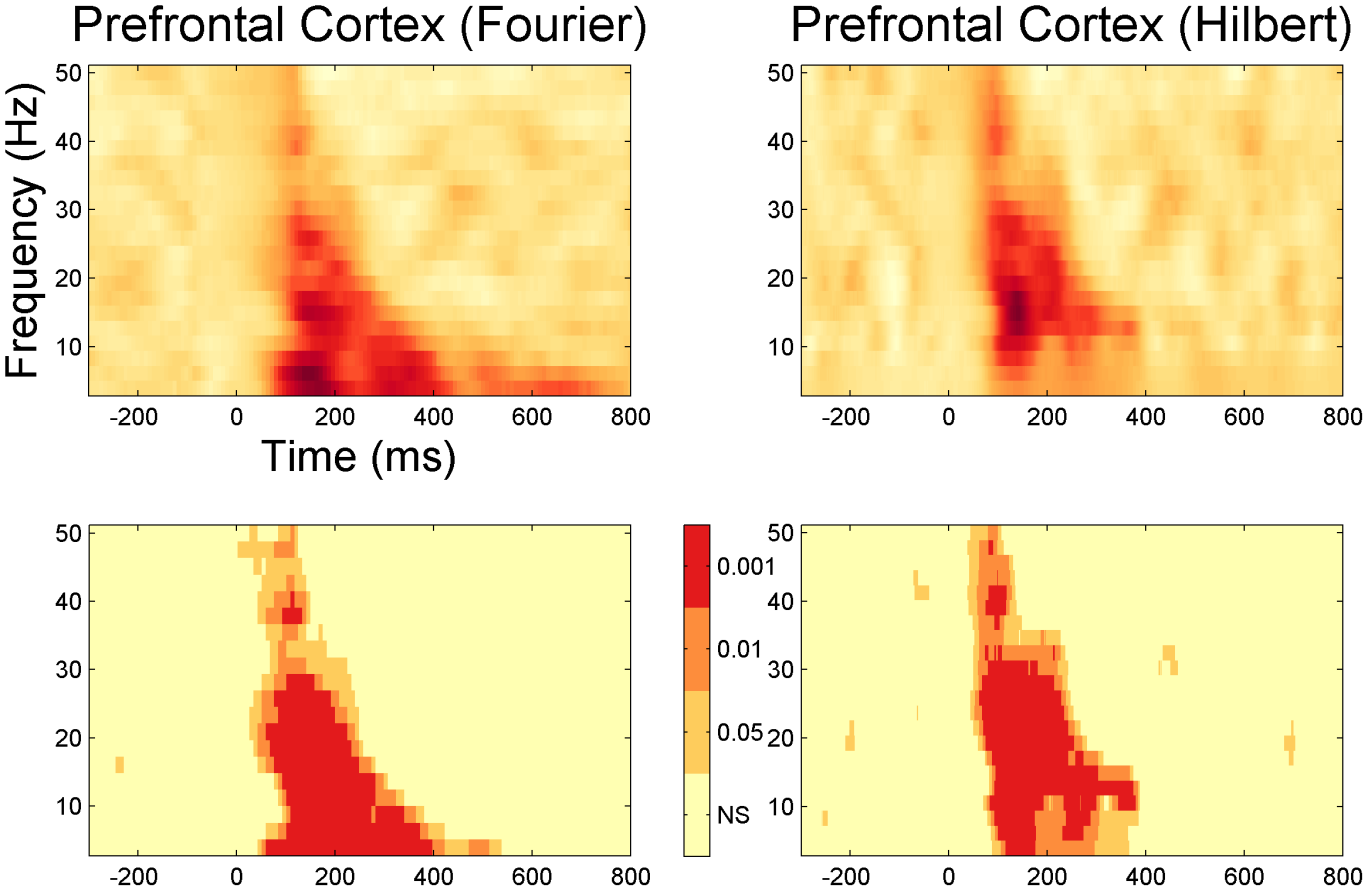
Average TF-maps (LICI^DFT^ and LICI^HT^) across channels and subjects. Each map was originally computed from the average trial (evoked response). The stimulation was applied over the DLPFC. The bottom panels show the statistical significance of the corresponding top graph at various levels of alpha: 0.001, 0.01, and 0.05.

**Figure 3:**
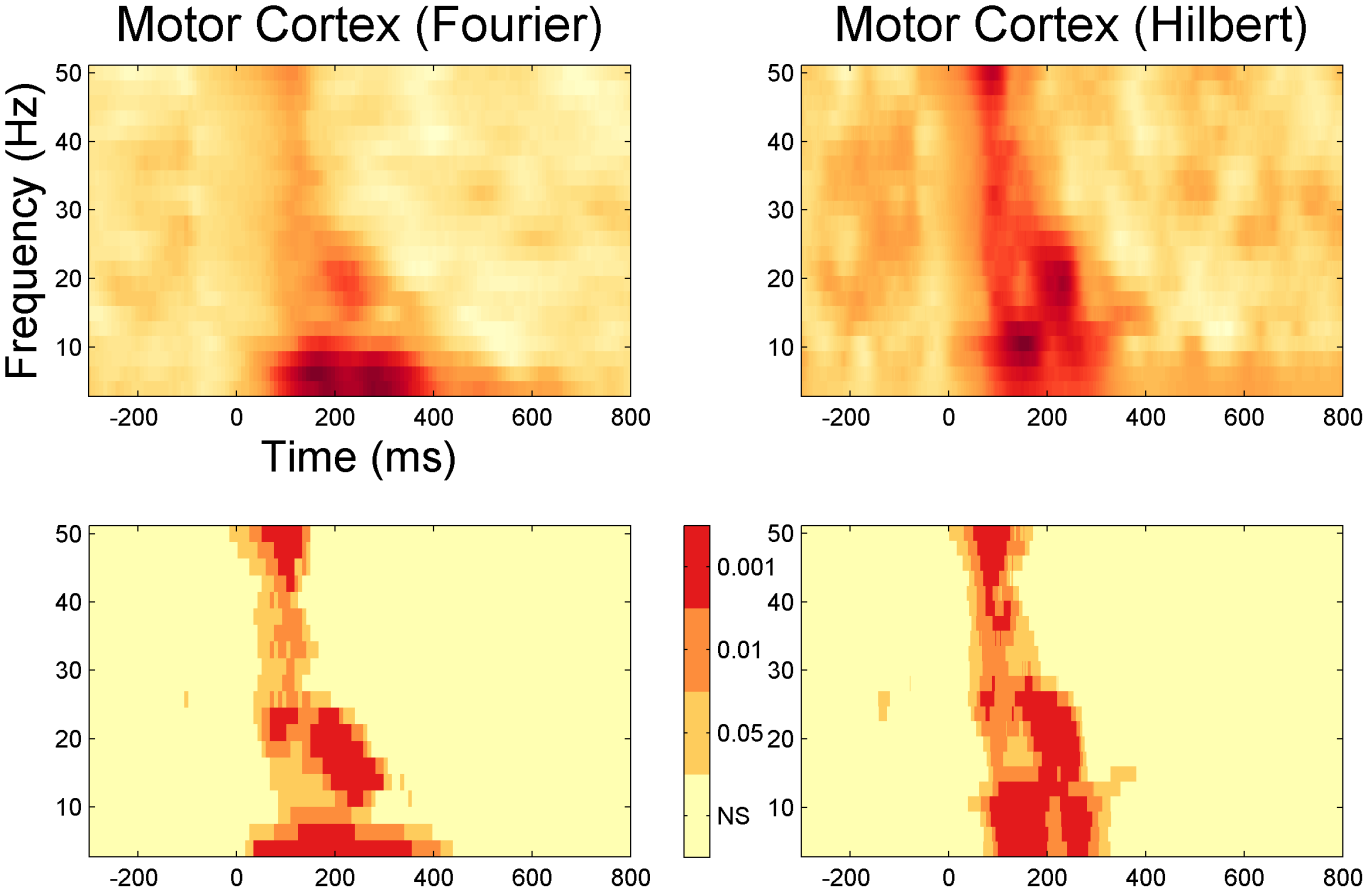
Average TF-maps (LICI^DFT^ and LICI^HT^) across channels and subjects. Each map was originally computed from the average trial (evoked response). The stimulation was applied over the MC. The bottom panels show the statistical significance of the corresponding top graph at various levels of alpha: 0.001, 0.01, and 0.05.

The evoked response corresponds to the activity that is phase-locked to the stimulus onset, that is, the activity that is enhanced by averaging across trials. TF-maps of LICI^DFT^ and LICI^HT^ where obtained from the evoked activity, by further averaging also across channels and participants. Figures 2 and 3 shows the TF-map of the LICI^DFT^ and LICI^HT^ in both DLPFC and MC. Positive values imply that the unconditioned evoked response is higher than the conditioned one. Consequently only sufficiently large positive values indicate inhibition.

How is “sufficiently large” defined? The statistical significance of the patterns on the top row of each figure is depicted in the bottom row. Three different alpha levels are shown, 0.05, 0.01 and 0.001. These levels are the corresponding percentiles (95, 99 and 99.9%) of a null distribution obtained by 1,000 random permutations of the labels corresponding to the unconditioned and conditioned pulse within each subject. In general, differences cluster around the period from 100 to 400 ms for DLPFC and 100 to 300 ms for MC. The areas of significant inhibition across the TF space were similar for both stimulation sites with lower frequencies tending to peak at a later time and for a wider period than higher ones.

### The strength and size of inhibition

The two bottom figures in 2 and 3 present the result of multiples hypothesis tests. From these graphs it cannot be concluded that inhibition or its effect is particularly large. In order to assert or disprove this claim, we first have to correct for multiple comparisons. That is, find a single statistic that summarizes the strength or size of the pattern we observe. In this case, we characterize the inhibition strength by the sum of the t-scores between the unconditioned and conditioned TF-map within a given TF-cluster. Similarly, the size can be characterized by the total number of significant time-frequency points within a given cluster. This parameter can also be computed for the population of random TF-maps and then compared with the original one. These tests are described in the method section. They control for multiple comparisons since only a single statistics is computed for each TF-map.

The result of applying these test to both DFT and HT show that no other TF-map in the population of random permutation is bigger in strength and size than the original population. Since this null hypothesis population consist of 10,000 samples, the result is consistent with a p-value <0.0001.

### The time course of inhibition

**Figure 4:**
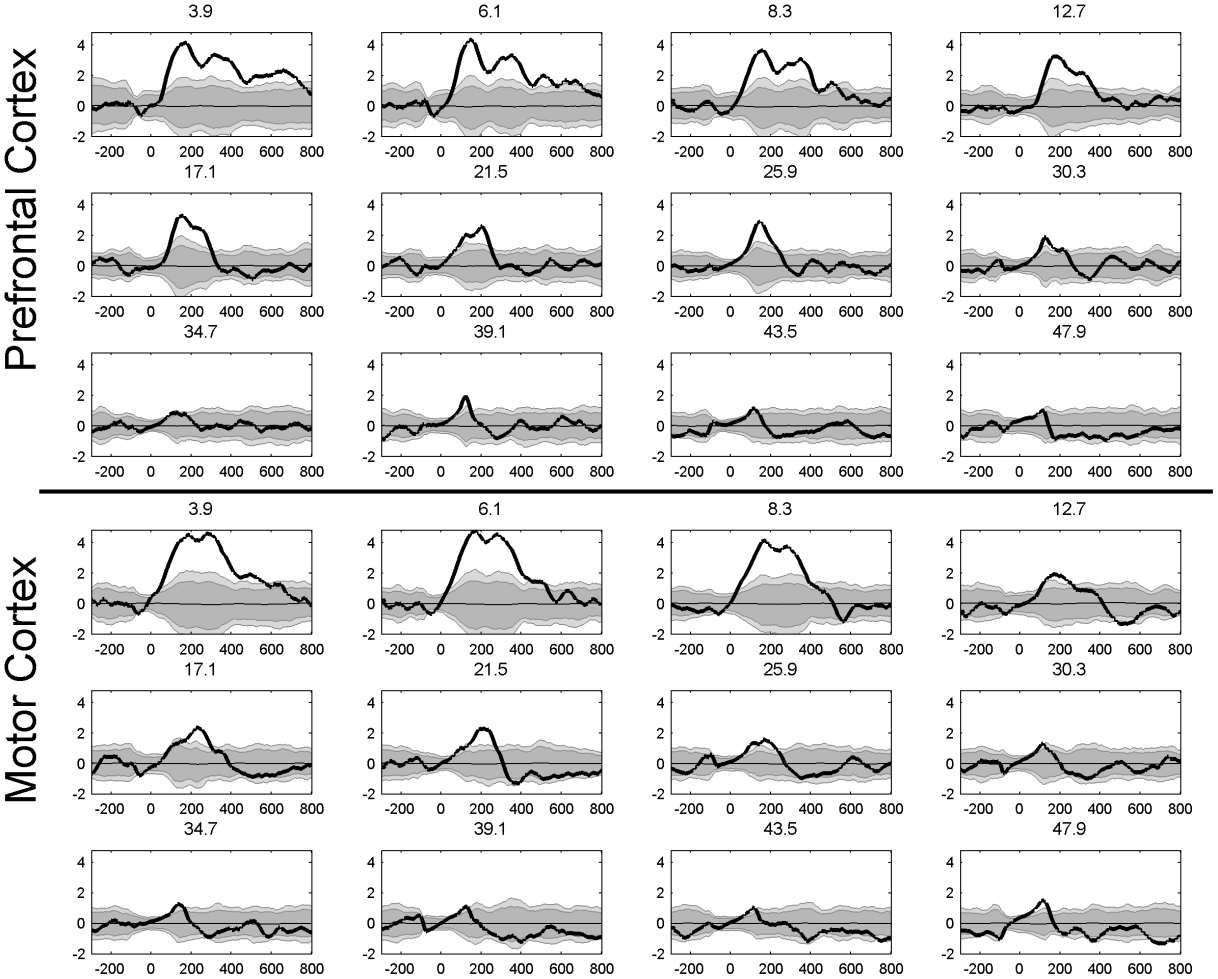
Time course of the LICI^DFT^ for the evoked activity averaged across all channels for 12 frequencies, from 3.91 to 47.9 Hz (bold line). The gray area depicts confidence intervals calculated as percentiles from the histograms produced by 1,000 random permutations of U- and C-conditions, dark grey (95%) (p<0.05) and light grey (99%) (p<0.01). The thin line corresponds to the mean value across all permutations.

**Figure 5:**
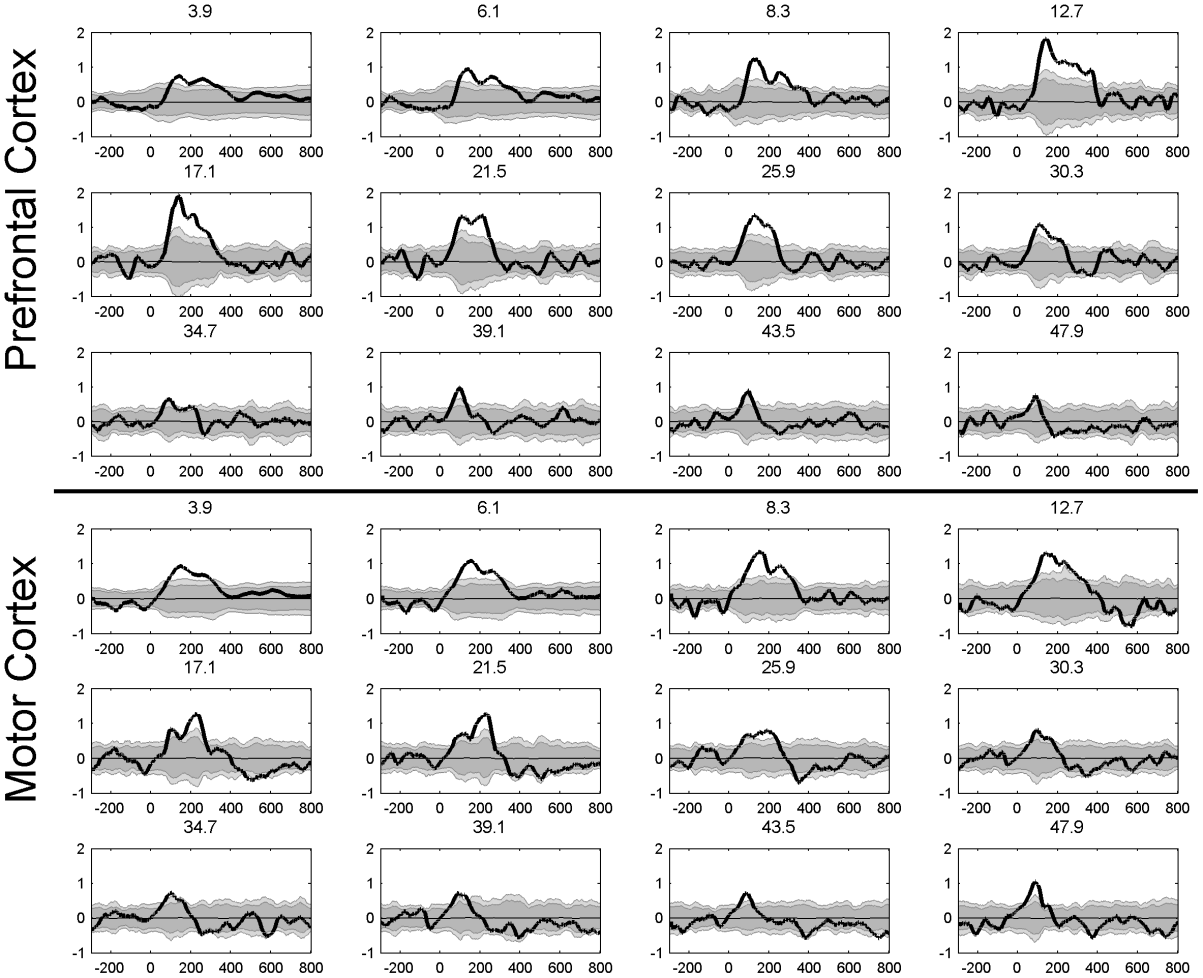
Time course of the LICI^HT^ for the evoked activity averaged across all channels for 12 frequencies, from 3.91 to 47.9 Hz (bold line). The gray area depicts confidence intervals calculated as percentiles from the histograms produced by 1,000 random permutations of U- and C-conditions, dark grey (95%) (p<0.05) and light grey (99%) (p<0.01). The thin line corresponds to the mean value across all permutations.

Figures 4 and 5 shows the time course of inhibition at different frequencies. This is a closer look at the temporal dynamic of the LICI^DFT^ and LICI^HT^ to that already presented in figures 2 and 3. The purpose of these plots is to take a closer look at the latency of the maxima and time course of inhibition. Only 12 frequencies equally spaced from the whole domain are depicted for each condition. Notice the extent of the area of significant values above the 99 percentile, both in amplitude and temporal coverage, and also the similarities between the HT and DFT analysis. Typically lower frequencies show a greater and wider extent of inhibition.

### Inhibition across channels

**Figure 6:**
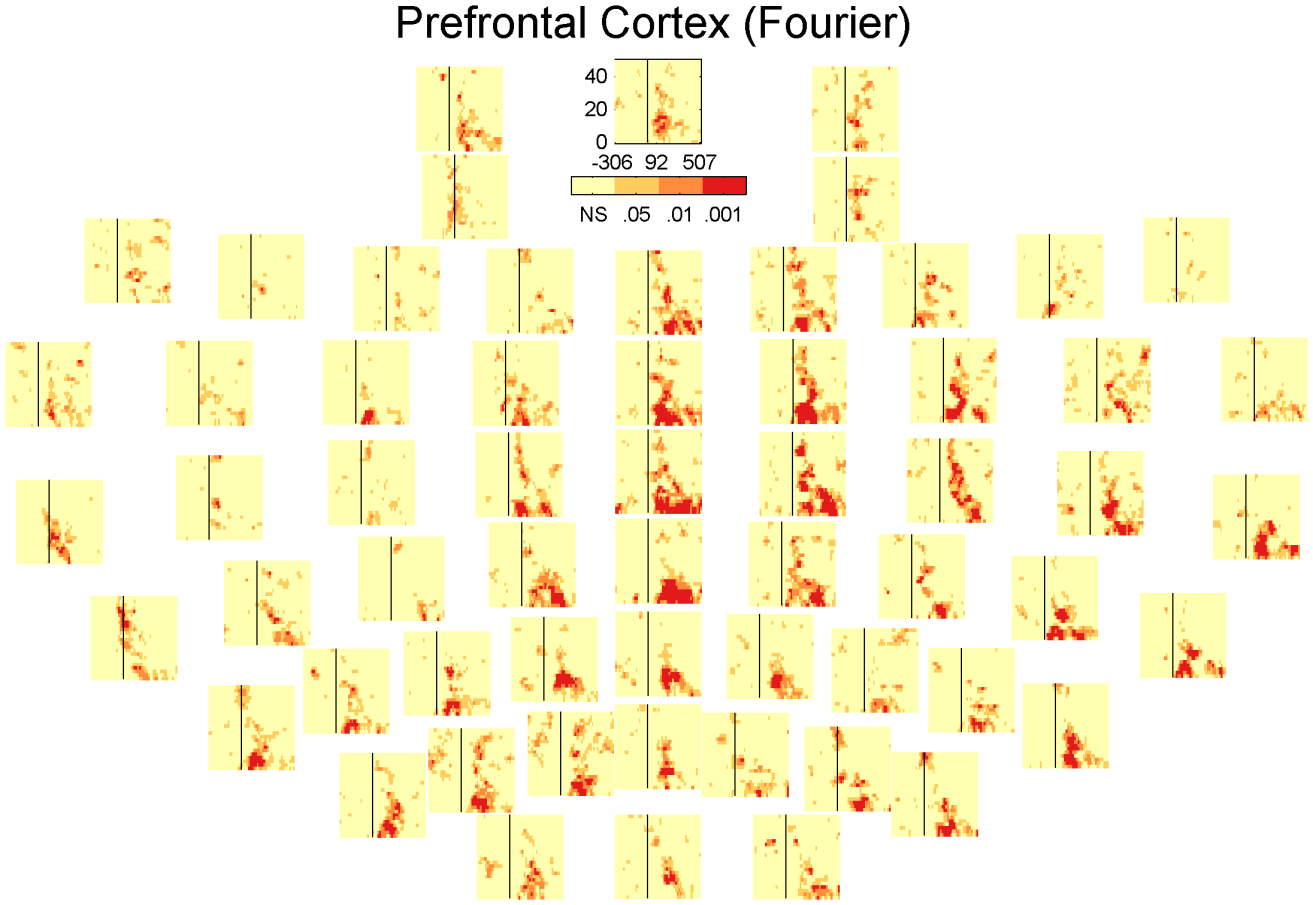
Statistical significance of each sample in the TF-maps of LICI^DFT^ for DLPFC corresponding to each specific electrode.

**Figure 7:**
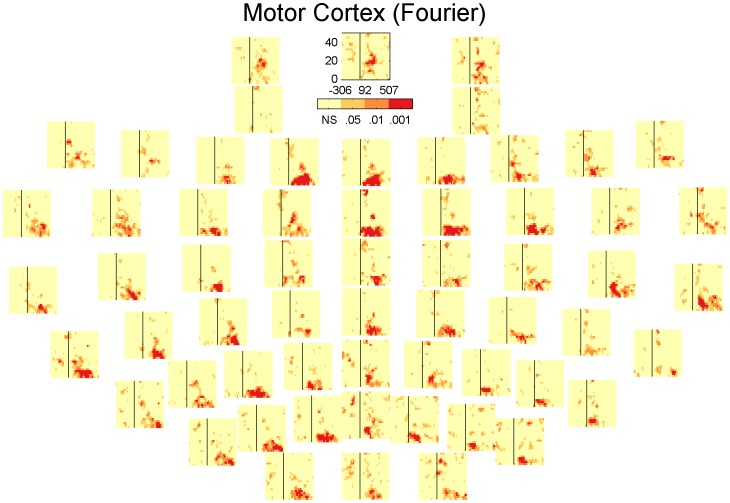
Statistical significance of each sample in the TF-maps of LICI^DFT^ for MC corresponding to each specific electrode.

**Figure 8:**
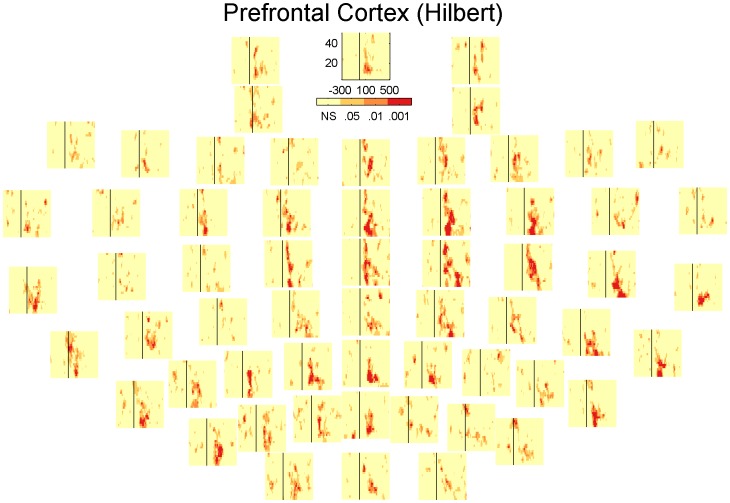
Statistical significance of each sample in the TF-maps of LICI^HT^ for DLPFC corresponding to each specific electrode.

**Figure 9:**
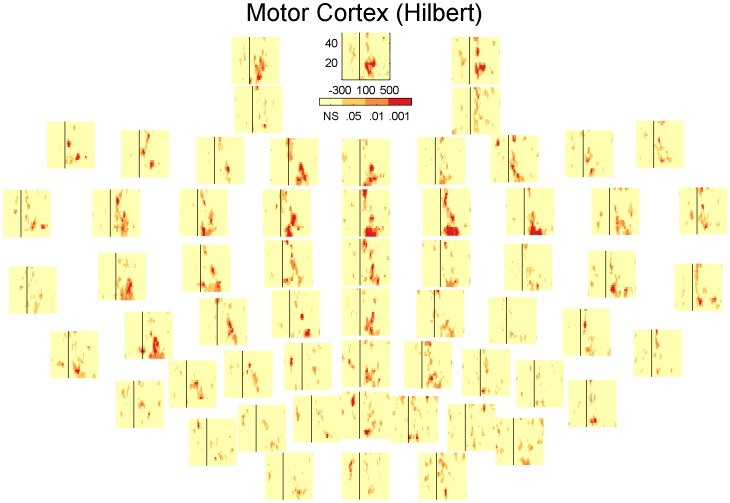
Statistical significance of each sample in the TF-maps of LICI^HT^ for MC corresponding to each specific electrode.

**Figure 10:**
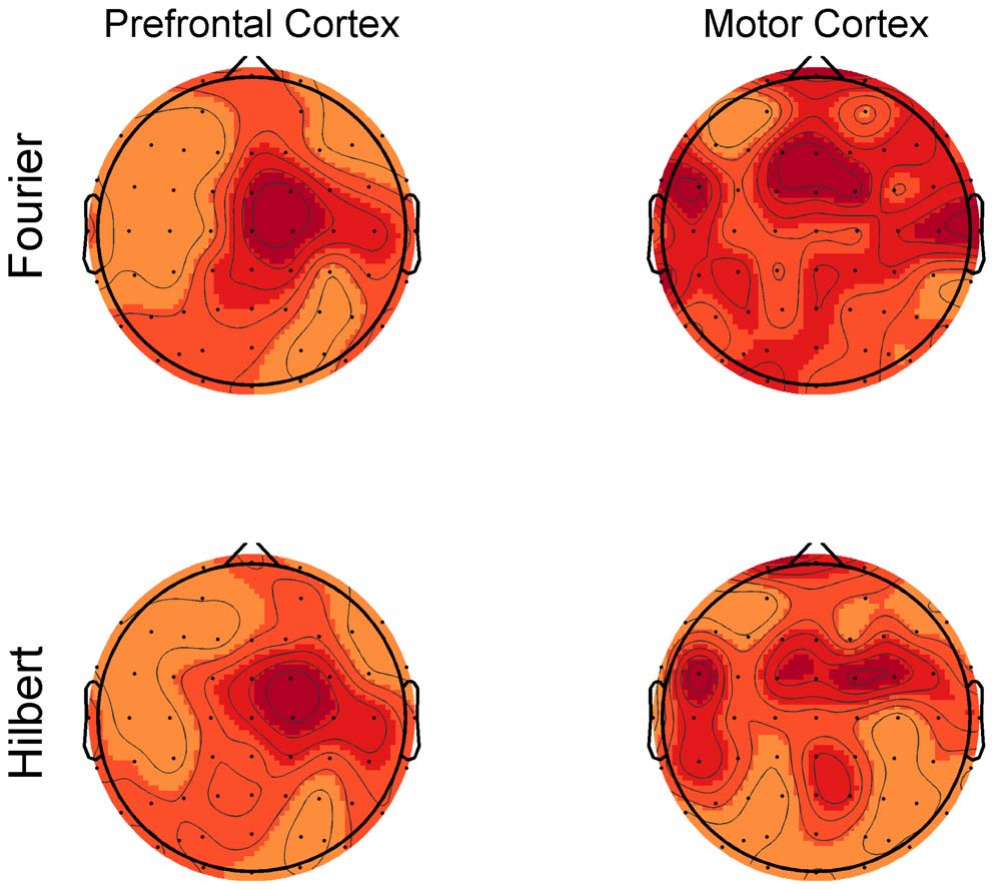
Strength of inhibition by electrode. Each value consists of the sum of all t-scores of LICI (DFT and HT), values within the major cluster of inhibition in the TF-map for each electrode within each specific condition (method and area of stimulation). These plots do not necessarily reflect the intensity of LICI at any specific time windows and, in this sense, are not directly comparable to previously published topographic maps of LICI.

In the results presented so far the channels are always averaged together. However the presence and extent of the inhibition could potentially vary as a function of the electrode position. Figures 6, 7, 8 and 9 show the significant activity from each channel in the TF-domain. An interesting observation in these plots is that in the DLPFC, figures 6 and 8, inhibition is relatively low over the whole TF-space in the channels close to the site of stimulation but is particularly strong over the central, midline channels. In the case of the motor, figures 7 and 9, inhibition does not seems to be particularly related to the site of stimulation either but is could be particularly strong on the contralateral site (figure 9). Figure 10 summarises figures 6, 7, 8 and 9 where each electrode is the sum of the t-scores over the larger significant cluster in the corresponding TF-map.

### The response pattern

**Figure 11:**
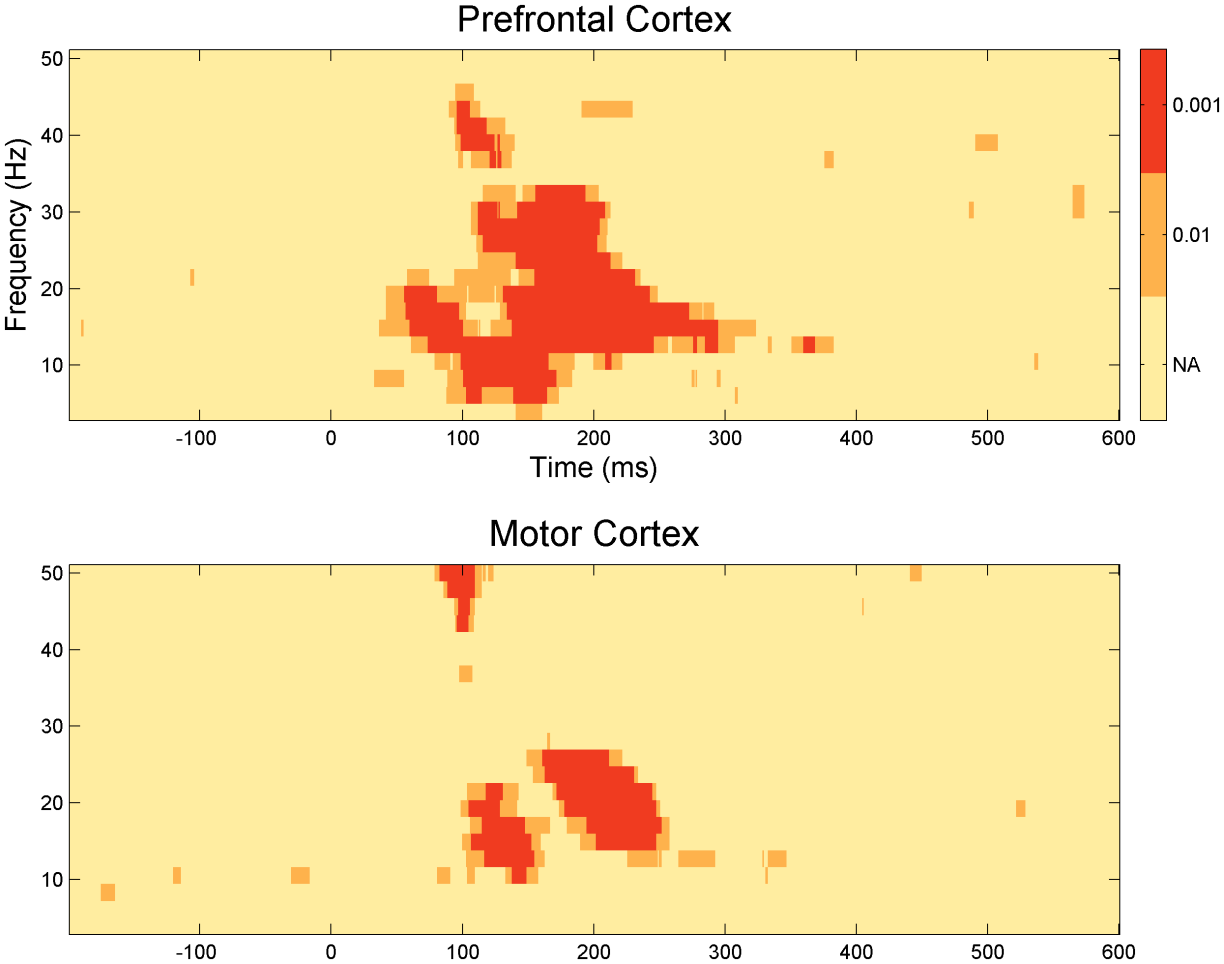
Significance levels of the differential response in pattern between the conditioned and unconditioned response. These plots are derived from the HT analysis.

So far we have only presented analyses on the relative strength of the activation between the conditioned and unconditioned response. The results clearly show a lower activation of the conditioned response. But, does this simple picture explain the differences between these two conditions? Can we tell anything about differences in the circuitry involved? EEG recordings do not possess a fine spatial resolution and comparisons between conditions at specific electrodes can be challenging in the presence of inter-subject variability in their response's topological map. Still, we can make such comparisons. The trick is to use each topological map, defined for each frequency and time, as a vector of 60 components (electrodes) and measure differences between the direction of that vector for the conditioned and unconditioned response. The rationale is that any large enough difference in the relative angle between the unconditioned and conditioned topo-vectors, correspond to differences in the spatial organization (pattern) of the two responses. Figure 11 shows the regions in the TF-space where differences in pattern are statistically significant. The test in this case also proceeds by 10,000 permutations across conditions. See a complete explanation in the material and methods section.

Similarly to figures 2 and 3, figure 11 presents multiple comparisons. This time we also have to correct for it if we intend to make the summarizing claim that single and paired evoke different spatial pattern of activity. After using random permutation of conditions we could obtain that, in fact, single and paired responses are highly different in this regard too, no permutation of condition showed a larger cluster of pattern differences than the actual conditions (p<0.0001).

## Discussion

The motivation of our analysis was twofold, to extend our previously presented tools and methodologies for the analysis of LICI and to pursue an exploratory analysis on the signature of LICI over an extended domain highlighting the main differences between DFT and HT. We illustrated the effect of using two alternative ways of calculating inhibition with the DFT and the HT methods. We report that LICI is a robust phenomenon with a p-value <0.0001, and that its size in the time frequency domain is also particularly wide (i.e., p<0.0001), in both cases, regardless of the method to estimate amplitude (Section 3.1). We also found that the spatial pattern of activation resulting from the single and paired stimulation differ statistically as strongly as the magnitude of inhibition (p<0.0001) (Section 3.4). Lastly, we demonstrated how inhibition is structured by slicing the results in its different dimensions, time, frequency, and space as shown in figures 2-9 (Sections 3.1 to 3.3).

In agreement with previous studies we have identified differences between the conditioned and unconditioned response with CI. However, this time we have analyzed a larger temporal window in which these differences may be more a consequence of inhibition rather than being part of the inhibitory process itself. In other words, observing large differences over roughly 300 ms (figures 2 and 3) between the activity generated by two stimulations should not lead us to conclude that the transient alteration of the postsynaptic threshold supporting CI has a longer duration than previously thought. If this were the case, the immediate implication is that there are additional mechanisms mediating LICI that are independent of GABA_B_ receptor-mediated inhibitory neurotransmission. Actual inhibition mediated by the release of GABA_B_ and subsequent generation of inhibitory post-synaptic potentials (IPSP), can still occur over a much shorter time scales (i.e., the first 100–150 ms after the test pulse). However, the effect of such transient inhibition can resonate over a much larger time scale. The particular spatial signature of LICI and its evolution in time, as we have described here, should be more related to the circuitry most affected by the initial GABA_B_-mediated inhibition and to the extent the unconditioned response still reverberates beyond the conditioned one.

Differences in the two analyses DFT and HT are expected within a certain degree. The DFT analysis show averages over a sliding window of 256 ms at specific frequencies, while the HT analysis deals with 4 Hz band-passed-pre-filtered time series. Variations in these settings are allowed and most likely it is expected that reducing the filter bandpass should produce better agreements with the DFT based analysis. We noted that differences between both analyses were in the delta and theta range, the bands of higher amplitude where it is most likely for the HT bandpass to produce more sensitive disagreements. However, taking into consideration the inherent nonstationarity of EEG we favor the HT analysis that produces instantaneous measures of phase and amplitude over the DFT-time averages. Also, the bandpass and center frequency of the HT can be adapted to cover specific frequency bands if needed, such as the alpha band. The degree of statistical significance in the result obtained by both analyses was very similar. Thus, choosing one or the other should not affect these findings.

These two time-frequency analyses are a natural and adequate choice to explore inhibition and its consequences. They have two main advantages over the tools used in previous studies. Firstly, they are transparent to the phase of the signal. Since it is based on the Fourier transform and the complex analytical signal, the phase can be discarded from each complex component keeping only the complex amplitude, which is a direct measure of activation. Consider this, in a scalar time series, the amplitude and phase are mixed together. That is why some of the analysis on ERP differences across conditions are based on measures of specific “landmarks” such as peaks, the only way to remove or control the phase component (since, at a peak, arguably, all ERPs have the same phase). Then, differences or latencies can only be evaluated at those specific time points. Secondly, the spectral analysis can be performed over a sliding time window or continuously, as in the case of the HT, which allows us to follow the evolution of the activation and to be able to address questions such as the location of maximal inhibition and its duration.

Although our analysis was mainly exploratory we addressed specific questions about the nature of the conditioned and unconditioned response's discrepancy. In particular we sought to investigate whether there were differences in amplitude as well as in the spatial organization of the response. We approached both cases by presenting descriptive TF-maps and topographic plots, as well as a rigorous statistical analysis that corrects for multiple comparisons. The statistical analysis described in this paper, the specific applications of the cluster mass test, produced particularly sensitive results without assumptions over the null distribution. This non-parametric test is based on random permutations of conditions across subjects. It corrects for multiple comparisons by reducing the whole TF-domain to a single number that accounts for the size of the main cluster of inhibition. The test revealed strong differences (p<0.0001) between the conditioned and unconditioned responses, in both magnitude and spatial organization, a strong indication of inhibition after considering comprehensibly, the whole time-frequency domain following the onset of the stimulation. We suggest that the reason no previous result showed such large LICI effects was partially due to the limited domain inspected in previous studies.

To our knowledge, no previous studies compared statistical differences in sensor space pattern by using the included angle between topographic-vectors as a measure of distance. The advantage of this procedure is that it clearly separates our notion of pattern from its intensity. This is an interesting approach when the main question is whether two conditions are different with regard to their specific pattern of activation of neuronal generators. Using this procedure, not every real difference is necessarily revealed (for the same reasons the inverse problem is ill defined, or more intuitively, that a 2D-shadow cannot fully determine a 3D-object; the topographic-vector is the 2D-shadow of the actual 3D positioned neural generators). However, the converse is true: differences detected by the analysis correspond to real differences in the temporospatial distribution of neuronal generators (the same shadow may correspond to different objects, but different shadows always correspond to different objects). This analysis adds new elements to our current understanding of the nature of LICI. We knew the conditioned-test paired response was a suppressed form of the test response alone. We now show that not only do they differ in the degree of activation; they seem to involve a different global circuitry. This result is not unexpected. Due to the typical non-linear nature of the brain response to stimulation it is natural to think that the inhibition induced by the conditioning pulse creates a sort of uneven spatial and temporal landscape for the next stimulation.

Our analysis presents a wider picture of LICI, in which the extent of the estimated CI in magnitude and time span is larger than previously documented. We have challenged ideas that somehow were an implicit natural extrapolation of previous more local analyses: that the extent of the measured inhibition is short in duration, that it is mostly restricted in strength to the site of stimulation, and that it is simply about amplitude differences in the response. We noticed that the size of the period of inhibition is strongly associated to the frequency, being particularly broad at lower frequencies. The location and strength of the peak of inhibition not only depends on the frequency but also the stimulation site. Figures 6, 7, 8, 9 and 10 reveal that the inhibition is a widespread phenomenon with no simple spatial structure, which could be partly the result of lack of signal propagation due to local inhibition. In fact, we also showed that this non-homogeneous spatial structure is, to some degree, due to differences in the temporospatial distribution of neuronal generators between the unconditioned and conditioned responses. Our results can have an important practical transcendence: that inhibition can be indexed, with high sensitivity, from areas distant to the stimulation site, mainly over central and contralateral channels. While GABA_B_-mediated inhibition can be local in nature, its effect can potentially amplify as we move away from the stimulation site in both time and space (in some directions). Thus, it is understandable that indexing inhibition from distant sites can offer a more sensitive characterization of the phenomenon, equivalent to what happen when perturbing, locally, a complex non-lineal system. This fact is particularly convenient from a practical standpoint. Understandably the electrodes closer to the site of stimulation are more affected by coil-induced artifacts, and thus the least reliable. This could conceivably help to explain why inhibition was not particularly strong over the area of stimulation (figures 6, 7, 8 and 9). Additionally, distant inhibition has been reported as interhemispheric inhibition and is mechanistically similar to LICI [23].

In the analysis presented in this study, the multiple comparisons correction is achieved on the time-frequency domain after averaging all channels, whereas the impact of each channel is addressed in a separate analysis. For future work, we recommend that the correction for multiple comparisons be directly assessed over the spatial-temporal-frequency domain. This is a more complex analysis but provides a more unified picture of LICI in its multiple dimensions. We also recommend that, in addition to DFT or HT, the wavelet transform be considered as another appropriate way to measure signal amplitude.

This study expands and develops further the initial reports of LICI by our group in concurrent scalp EEG. We consider the tools and statistical approach proposed in this paper to quantify LICI could be potentially accepted as a guideline. Attempts have been made to explore LICI using TMS-EEG in psychiatric disorders such as: schizophrenia and bipolar disorder [13] and psychopathic offenders [14]. These studies have shown some specific imbalances of inhibition that could become useful in building a future biomarker [24]. Within this line of research, the characterization of LICI that we have presented here can be used to enhance the pool of parameters that characterize the normal response to the TMS pulse. This pool or set of features offer a wider window to explore CI differences between populations. The statistical treatment presented in this paper can be easily adapted to the case of comparing clinical populations in which the permutation can be performed on group labels instead of condition labels. The novel analysis of statistical differences in pattern can also be used for comparing different populations or conditions. In this sense it could potentially uncover network differences between the normal and the pathological brain.
